# Distinct Facial Processing Related Negative Cognitive Bias in First-Episode and Recurrent Major Depression: Evidence from the N170 ERP Component

**DOI:** 10.1371/journal.pone.0109176

**Published:** 2014-10-14

**Authors:** Jiu Chen, Wentao Ma, Yan Zhang, Xingqu Wu, Dunhong Wei, Guangxiong Liu, Zihe Deng, Laiqi Yang, Zhijun Zhang

**Affiliations:** 1 Neurologic Department of Affiliated ZhongDa Hospital, Neuropsychiatric Institute and Medical School of Southeast University, Nanjing, Jiangsu Province, China; 2 Center for Mental Disease Control and Prevention, Third Hospital of the People’s Liberation Army, Baoji, Shaanxi Province, China; Cardiff University, United Kingdom

## Abstract

**Background:**

States of depression are associated with increased sensitivity to negative events. For this novel study, we have assessed the relationship between the number of depressive episodes and the dysfunctional processing of emotional facial expressions.

**Methodology/Principal Findings:**

We used a visual emotional oddball paradigm to manipulate the processing of emotional information while event-related brain potentials were recorded in 45 patients with first episode major depression (F-MD), 40 patients with recurrent major depression (R-MD), and 46 healthy controls (HC). Compared with the HC group, F-MD patients had lower N170 amplitudes when identifying happy, neutral, and sad faces; R-MD patients had lower N170 amplitudes when identifying happy and neutral faces, but higher N170 amplitudes when identifying sad faces. F-MD patients had longer N170 latencies when identifying happy, neutral, and sad faces relative to the HC group, and R-MD patients had longer N170 latencies when identifying happy and neutral faces, but shorter N170 latencies when identifying sad faces compared with F-MD patients. Interestingly, a negative relationship was observed between N170 amplitude and the depressive severity score for identification of happy faces in R-MD patients while N170 amplitude was positively correlated with the depressive severity score for identification of sad faces in F-MD and R-MD patients. Additionally, the deficits of N170 amplitude for sad faces positively correlated with the number of depressive episodes in R-MD patients.

**Conclusion/Significance:**

These results provide new evidence that having more recurrent depressive episodes and serious depressive states are likely to aggravate the already abnormal processing of emotional facial expressions in patients with depression. Moreover, it further suggests that the impaired processing as indexed by N170 amplitude for positive face identification may be a potentially useful biomarker for predicting propagation of depression while N170 amplitude for negative face identification could be a potential biomarker for depression recurrence.

## Introduction

Depression is a commonly occurring mental disease [1]. The first study of the cognitive theories of depression [2] indicated that cognitive processing can be affected by the unconscious negative or pessimistic schemata activated by stressful events, which include selection, encoding, perception, and interpretation of actual experiences [3], [4]. The depressive effect is thought to be due to the negative schemata, which lies dormant until activated by stressful life events [2], [4]. However, previous studies have demonstrated that negative cognition comes into being only during depressive episodes [5], and the cognitive processing bias for negative stimuli plays a key role in its onset [6]. Other supporting evidence suggests that depressive states are classically related to increased sensitivity to negative events [7]. This hypersensitivity may be further enhanced with each recurrent depressive episode.

Gotlib and Neubauer have demonstrated that these negative schemata influence information processing by elevating the salience of negative events and by decreasing the salience of positive events [8]. Accordingly, for positive social stimuli, patients with major depression DISORDER (MDD) were less likely to identify mild happy expressions as more intense than neutral and negative expressions, relative to controls [9]. Using emotional stimuli, numerous studies have indicated that patients with MDD have an attentional bias that is specific to sad faces and an impaired inhibition of attending to negative social information [7], [10], [11]. However, little is known about how effective facial emotional stimuli are during perceptual processing and whether they may lead to a better understanding of mood-related attention bias in depression, particularly in recurrent depression. An in-depth understanding of the specific time course of cognitive processing during the perceptual processes of emotional stimuli can help to describe which specific cognitive processes are influenced by mood-related biases.

Recently, a study from the Canadian National Population Health Survey has reported that the recurrence of major depressive episodes strongly depends on the number of previous episodes [12]. Several pieces of neuroimaging evidence also suggest that the altered striatal connectivity may be affected by the number of depressive episodes, thus contributing to depressive recurrence risk [13]. First episode major depression patients (F-MD) had smaller left hippocampal volumes, left-right asymmetry [14] and larger amygdala volumes [15]. Numerous cross-sectional epidemiological studies have shown that the severity of depression is positively associated with the number of episodes, and that stressful life events during mild and long-term periods may reinforce depressive recurrent risk [16], [17]. Previous studies have indicated that recurrent major depression patients (R-MD) have more serious cognitive impairment compared to F-MD patients. Examples of such cognitive impairment are autobiographical memory [18], verbal memory performance [19], executive function [20] and mental representation processing [21]. Also, recurrence chronically modifies access to emotive memories [18]. Moreover, previous studies have demonstrated that R-MD patients have an increased oxidative stress [22] and higher serum neopterin levels [23] compared to F-MD patients. Taken together, the evidence from these studies suggest that R-MD patients present with a more serious impairment compared with F-MD patients, and the recurrence of depressive episodes may reinforce the damage severity. Very little is known, however, about the relationship between the abnormal neural processing of emotional facial expressions and the number of depressive episodes. Furthermore, the differences between cognitive processing biases for negative faces between F-MD and R-MD patients are still poorly understood.

Event-related evoked potential (ERP) measurements, a powerful non-invasive approach that have a time resolution in the millisecond range and allow assessment of cognitive brain function, have been widely used to investigate individuals’ information processing of different cognitive schemata [24]. ERP measurements are a type of long-latency evoked potentials extracted from ongoing electrical cerebral background activity by averaging related procedures to reflect human information processing [25]. In accordance with these experimental manipulations, the measurements can then identify and characterize impairments that may exist in pathological states. The amplitude of the electrophysiological response reflects the intensity of the internal information processing, while its latency represents the timing of that process.

Different electrophysiological components are considered to be associated with different cognitive functions. The N170 component, which is a negative-going component and arose from occipito-temporal brain generators, was first reported by Bentin et al. (1996) [26]. Subsequently, numerous ERP studies have demonstrated that the N170 component is sensitive to facial emotional expressions. For example, Batty and Taylor used unfamiliar faces expressing the six basic emotions and neutral faces, and showed that ERP measurements report global effects of emotion from 90 ms, while latency and amplitude differences for emotional expressions are found from 140 ms. This suggests that the N170 component may represent rapid processing of emotional expressions [27]. Using an emotional faces task, Blau et al. and Japee et al. demonstrated that the N170 response shows a strong modulation by emotional facial expression [28], [29]. Recently, Wronka and Walentowska used an emotional faces task to discriminate emotional expressions and also demonstrated that N170 amplitude was modulated by facial emotional expressions [30]. Furthermore, in these previous studies, all of the researchers have consistently indicated that if the faces are presented as the attentional focus and the subjects are required to direct their attention to the facial expressions, N170 amplitude is modulated by facial emotional expressions [31]–[34]. Taken together, the evidence from these studies shows that the N170 amplitude and latency modulation can be used as a neurophysiological indicator of the cognitive processing of emotional faces. Moreover, the onset of the N170 effect can be used as a chronopsychophysiological marker for the onset of the processing of emotional expressions. To sum, the N170 component is an ideal brain marker to assess possible cortical markers of emotional face processing in F-MD and R-MD patients.

The objective of our current study is to compare the neural processing of emotional facial expressions by patients with a first episode and recurrent depression to that of healthy control subjects using the ERP technique. Based on the previous studies, we predict that patients with MDD will present with an impairment of emotion processing. We also predict that there will be a difference between F-MD and R-MD patients. The F-MD group will likely have longer N170 latencies and lower N170 amplitudes to three emotional faces relative to the HC group. The R-MD group will likely have lower N170 amplitudes for happy and neutral faces, but higher N170 amplitudes for sad faces relative to the HC group. Moreover, we further predict that there will be a correlation between the number of depressive episodes and the altered processing of emotional faces. The new information we discover regarding the repeated physiopathologic mechanism for depression will be extremely valuable for clarifying diagnoses, advising disease treatments and planning clinical trials.

## Materials and Methods

### Ethics statement

All procedures were approved by the Human Participants Ethics Committee of the Baoji Third Hospital of the People’s Liberation Army and written informed consent was obtained from all participants prior to entry into the study. Ability to provide informed consent was assessed first by the participant’s referring clinician who was not associated with the study and an additional study physician prior to inclusion in the study. None of the participants had significant cognitive impairment which would interfere with their ability to provide informed consent. All potential participants who declined to participate or otherwise did not participate were eligible for treatment and were not disadvantaged in any other way by not participating in the study.

### Subjects

From inpatients (all of whom were Chinese Han and right-handed) at Center for Mental disease Control and Prevention of Baoji Third Hospital of the People’s Liberation Army in China, we recruited 45 F-MD patients (21 males and 24 females) and 40 R-MD patients (18 males and 22 females); see Table 1 (subjects were aged 18–65 years; mean age: F-MD group: 30.6±11.3 years; R-MD group: 32.8±13.6 years). Psychiatric diagnoses were determined by at least two psychiatrists who agreed on the diagnosis based on the DSM-V criteria [1] for major depression.

**Table 1:** Demographics and clinical measures of depressed patients and HC subjects.

	F-MD	R-MD	HC		
Items	(N = 45)	(N = 40)	(N = 46)	*F* values (χ^2^)	*p* values
Age (years)	30.6(11.3)	32.8(13.6)	31.1(10.8)	0.736	0.830
Gender (males/females)	21/24	18/22	22/24	0.043	0.214
Education (years)	12.6(3.0)	13.1(3.3)	13.8(2.1)	1.003	0.672
HDRS_17_	22.6(7.9)^b^	23.8(8.9)^c^	2.5(1.3)	8.322	0.010*
MMSE scores	25.1(1.3)^a b^	21.5(2.5)^c^	29.0(2.2)	6.242	0.013*
AVLT-DR	3.8(1.2)^a b^	1.9(1.5)^c^	7.8(2.3)	7.660	0.007*
TMT-A (seconds)	78.3(32.6)^a b^	88.6(38.3)^c^	67.4(16.4)	9.603	0.000*
TMT-B (seconds)	212.4(121.4)^a b^	258.6(143.2)^c^	175.3(63.2)	10.312	0.000*
SDMT	34.8(12.3)^a b^	23.9(10.6)^c^	39.6(13.2)	9.328	0.000*
DST	11.3(2.2)^b^	10.0(2.1)^c^	12.5(2.6)	4.463	0.031*
CDT	7.3(1.7)^b^	7.2(1.3)^c^	9.1(1.4)	3.204	0.040*
Number of episode	1.0(0.0)	3.6(2.0)	NA	NA	NA
First	45(100%)	-			
Second	-	16(40.0%)			
Third	-	10(25.0%)			
Fourth	-	8(20.0%)			
Fifth	-	5(12. 5%)			
Sixth	-	1(2.5%)			
Age at onset (years)	28.1(2.1)	28.3(4.6)	NA	NA	NA
Duration of illness (years)	0.6(0.3)^a^	3.5(1.1)	NA	NA	NA
Duration of current episode (weeks)	28.2(6.2)^a^	31.0(8.1)	NA	NA	NA
Antidepressant comedication	38(84.4%)	40(100%)	NA	NA	NA

Notes: Abbreviation: F-MD: first episode of major depression; R-MD: recurrent episodes of major depression; HC: Healthy controls; NA: not applicable; HDRS_17_: 17 items the Chinese Hamilton Depression Rating Scale; MMSE: Mini mental state exam; AVLT-DR: Auditory verbal learning test- delayed recall; TMT-A: Trail making test-A; TMT-B: Trail making test-B; SDMT: Symbol digit modalities test; DST: Digit span test; CDT: Clock drawing test.

*Significant differences were found among first episode depression patients and recurrent depression patients and HC subjects. *P* values were obtained by ANOVA analysis except for gender (chi square test). a–c: post-hoc analysis (LSD test for demographic information and Bonferroni correction for multiple comparison) further revealed the source of ANOVA difference (a: first episode patients vs. recurrent patients; b: first episode patients vs. HC subjects; c: recurrent patients vs. HC subjects).

Illness durations ranged from 2 months to 30 years (mean illness duration: F-MD group: 0.6±0.3 years; R-MD group: 3.5±1.1 years). Patients’ education ranged from 8 years to 22 years (mean years of education: F-MD group: 12.6±3.0 years; R-MD group: 13.1±3.3 years). The severity of depression was evaluated with the 17-item Hamilton Depression Rating Scale (HDRS) [35]. A minimum score of 22.4 was required to participate. All patients received the same antidepressant medication (serotoninergic antidepressive treatment) and the same psychological treatment (psychotherapy interviews and group therapy) [4]. All subjects were clinically stable at the time of testing.

For comparison, 46 healthy control subjects (HC, 22 males and 24 females) without any history of psychiatric illness were matched to the patients in the F-MD and R-MD groups according to age, gender and education (subjects were aged 18–65 years; mean age: 31.1±10.8 years). Exclusion criteria for the patients and the control subjects were a history of substantial head injury, seizures, neurological diseases, dementia, impaired thyroid function, corticoid use or alcohol or substance abuse or dependence. **Table 1** provided group information about age, gender, and education.

### Procedures

The experiment was performed with E-Prime 2.0 software (Psychology Software Tools Inc., Pittsburgh, USA). The experimental procedure used an ‘‘emotional oddball paradigm’’ [24], [25]. Stimuli consisted of six faces that were selected from a highly standardized set of pictures developed by the Psychology Department of the Chinese Academy of Sciences. The faces had neutral, happy and sad expressions [36]. Standard faces always presented neutral expressions, whereas deviant faces were either the same face displaying an emotion (happy or sad) or a different neutral face (change in identity).

Subjects were confronted with a total of 16 blocks that were defined by 100 stimuli (e.g., 80 frequent stimuli with face A neutral; five deviant face A happy, five deviant face A sad and 10 face B neutral). During the ERP recording, subjects sat on a chair in a quiet, dimly–lit, sound-proof room with their head restrained in a chin rest and placed at 1 m from the 17″ computer screen (refresh rate 75 Hz). Stimuli subtended a visual angle of 3°×4°. Similar to previous reports [37], [38], faces were presented for 100 ms in order to assure conscious perception of the faces. A black screen was displayed as an intertribal interval and lasted randomly between 1300 and 1600 ms. The subjects had 1500 ms to answer after the stimulation onset [25]. The participants had to quickly point out the occurrence of a deviant face among the presentation of standard faces by pressing a button with their right index finger. The order of the 16 blocks varied across participants. Reaction time (RT) and accuracy were recorded automatically for each trial.

### EEG acquisition and analysis

Electroencephalogram (EEG) data acquisition was carried out continuously throughout the experiment. The EEG data were acquired using a BrainAmp MR portable ERP system (Brain Products GmbH, Munich, Germany) with 32 scalp electrodes. Electrodes were placed according to the extended international 10–20 system [39]. Two ear electrodes served as reference electrodes in off-line analyses, and the AFz electrode was used for grounding. The vertical electro-oculogram (VEOG) and horizontal electro-oculogram (HEOG) were recorded with bipolar channels from sites above the midpoint of the left eye and 10 mm from the right lateral canthus in order to control the interference of eye blinks with the EEG-signal. The EEG was band-pass filtered from 0.1 to 100 Hz, amplified with a gain of 20 and data was stored on a computer disk at the sample rate of 500 Hz. The EEG signal was analyzed using Brain Vision Analyzer software (Brain Products GmbH, Munich, Germany). Offline, the signal was digitally filtered (high pass = 0.1 Hz, low pass = 30 Hz). EEG signals with amplitude larger than ±70 µV were interpreted as artifacts and rejected. To calculate the ERP, epochs of EEG were averaged off–line, and time was locked to stimulus onset from 200 ms pre–stimulus to 800 ms post stimulus relative to a 200 ms pre–stimulus baseline. Only trials leading to correct responses were included. The mean number of epochs included in each ERP average varied between 73.2 and 128.6 for the various types of stimuli used.

### Statistical analyses

The statistical analyses were conducted with SPSS 17.0 software (SPSS Inc., Chicago, IL, USA). The analysis of variance (ANOVA) and chi-square test were used to compare the demographic data and neuropsychological test performances between patients and HC subjects. Accuracy and RTs were also analyzed. After rejecting responses with RTs shorter than 200 ms and longer than 1200 ms, a mean RT of correct responses in each stimulus condition was calculated for each subject. The data for accuracy and RTs were analyzed by separate 3×3 ANOVAs with Group (F-MD, R-MD, and HC) as one between-subjects factor, target expression (happy, neutral, and sad) as within-subject factors.

The peak amplitude and peak latency of the N170 response were identified within a time window of 130–210 ms after the onset of the target stimulus [28], [40]. The quantification was restricted to the data from the lateral occipital channels P7 and P8. All ERP data were analyzed by a 3×3×2 repeated–measure ANOVA with Group (F-MD, R-MD, and HC) as one between-subjects factor, target expression (happy, neutral, and sad), and electrode location (P7 vs. P8 for N170) as within-subject factors. Post hoc comparisons were analyzed by Bonferroni test.

To investigate the behavioral significance of altered amplitude and latency of event-related N170 potential, the linear regression model with a step-wise analysis was used. This allowed us to examine the relationships between the amplitude and latency of N170, the HDRS_17_ score and behavioral data in each group, and between the amplitude and latency of N170 and the number of episodes in the R-MD group. The statistical significance threshold was set at *P*<0.05.

## Results

### Demographic and neuropsychological characteristics

Demographic characteristics were shown in **Table 1**. No significant differences in age, gender, and years of education were noted between all groups (all *P*s>0.05). Compared with HC subjects, the F-MD and R-MD groups showed the significant declines in muilt-domains of cognitive function, including episodic memory (i.e., AVLT -DR), executive function (i.e., TMT-A and -B), perceptual speed (i.e., SDMT), working memory (i.e., DST) and visuo-spatial cognition (i.e., CDT) (all *P*s<0.05). Compared with F-MD patients, R-MD patients showed significantly lower MMSE, AVLT -DR, SDMT, and higher TMT-A and -B scores (all *P*s<0.05).

### Behavioral results

#### Reaction times

There was a significant main effect of group in F-MD patients (mean = 1026±312 ms). The F-MD group was slower compared with both the R-MD (mean = 964±362 ms) and HC groups (mean = 842±252 ms) (*F* (2, 128) = 14.83, *P*<0.001). A significant main effect of target expression was also observed (*F* (2, 128) = 8.26, *P* = 0.030). The interaction of group×target expression was significant (*F* (2, 128) = 24.62, *P*<0.001).

**Figure 1:**
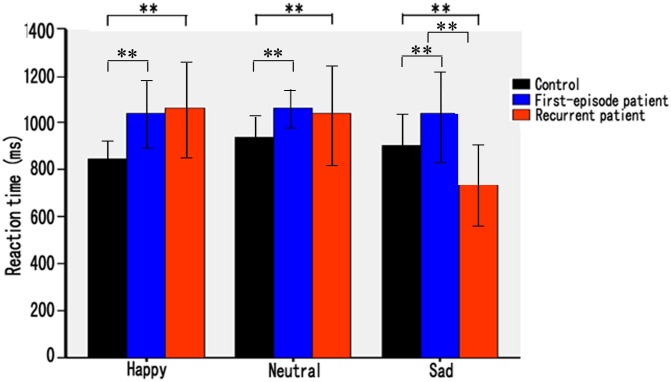
Mean reaction time (ms) for first episode and recurrent depression patients and healthy controls in happy, neutral, and sad face task.

Post hoc comparisons showed that F-MD patients had slower RTs for happy, neutral and sad faces relative to HC subjects (*t’*s>4.03, *P*’s<0.004). R-MD patients had slower RTs for happy and neutral faces, but shorter RTs for sad faces relative to HC subjects (*t’*s>6.23, *P*’s<0.001). R-MD patients also had shorter RTs for sad faces compared with F-MD patients (*t*(83) = 2.92, *P* = 0.005) (Figure 1).

#### Accuracy

There was a significant group effect on accuracy in R-MD patients (mean = 80.2±10.3%). Accuracy was lower than both the F-MD group (mean = 85.8±10.8%) and the HC group (mean = 92.7±5.2%) (*F* (2, 128) = 22.06, *P*<0.001). No significant main effect of target expression was found (*F* (2, 128) = 1.64, *P* = 0.063). The interaction of group×target expression was significant (*F* (2, 128) = 3.25, *P* = 0.024).

Post hoc comparisons showed that the F-MD and R-MD groups had lower accuracy for happy and neutral faces relative to HC group (*t’*s>3.26, *P*’s<0.008). However, the R-MD group had greater accuracy for sad faces compared with the F-MD and HC groups (*t’*s>2.32, *P*’s<0.016).

### Electrophysiological results

#### N170 amplitude

There was a significant main effect of group on lower amplitudes in the R-MD group (mean = –6.82±2.40 µV). Amplitudes were lower when compared with those of the F-MD (mean = –7.65±3.26 µV) and HC groups (mean = –10.16±2.82 µV) (*F* (2, 124) = 12.38, *P*<0.001). A significant main effect of target expression was observed (*F* (2, 124) = 9.63, *P*<0.001). No significant main effect of electrode location was found (*F* (1, 124) = 1.22, *P* = 0.096). The interaction of group×target expression was highly significant (*F* (2, 124) = 13.02, *P*<0.001).

**Figure 2:**
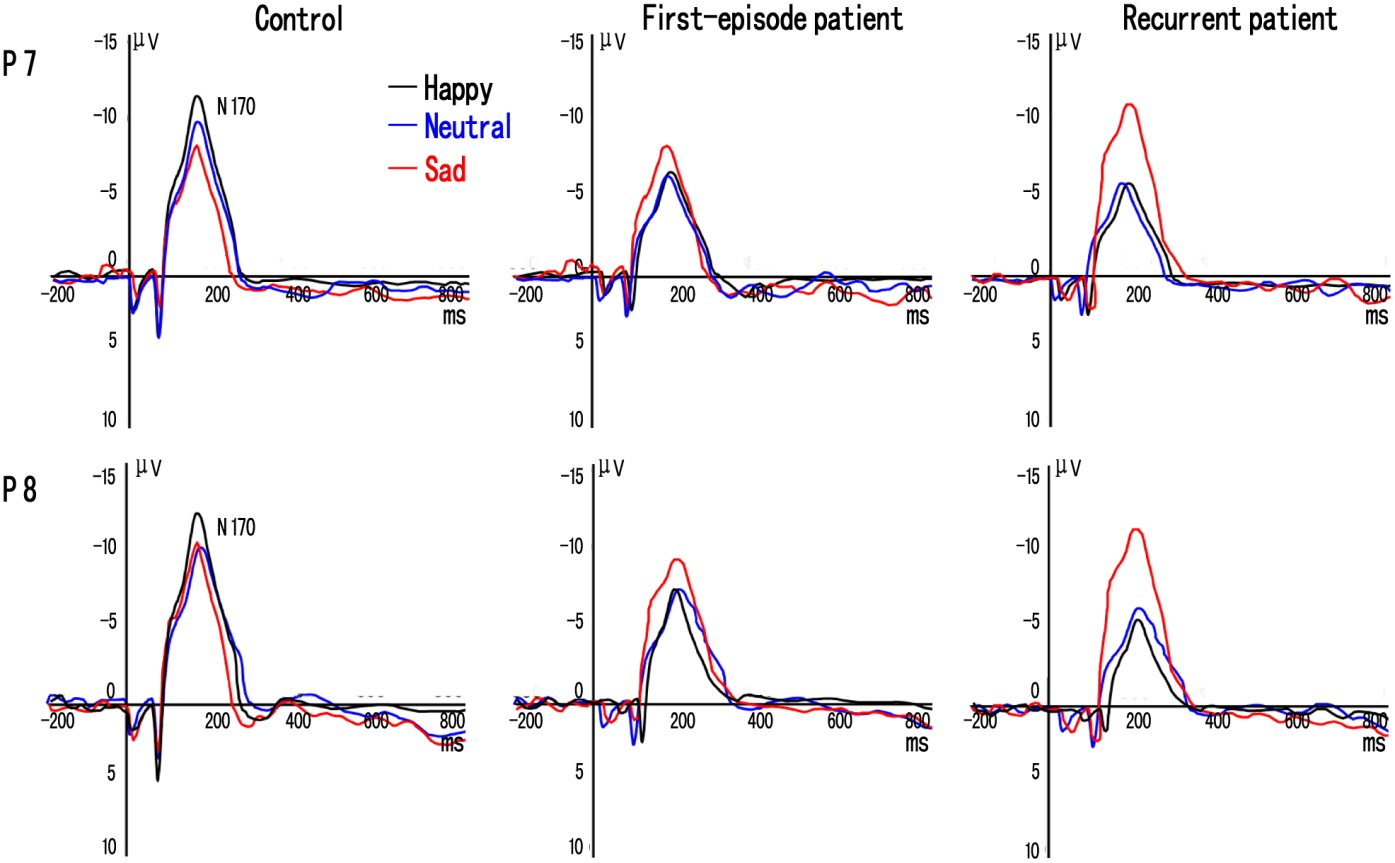
Grand-averaged event-related potential (ERP) waveforms of the N170 components elicited by happy (black line), neutral (blue line), and sad (red line) face pictures at P7 and P8 electrodes in first episode and recurrent depression patients and controls.

Post hoc comparisons showed that the F-MD group had lower amplitudes for happy, neutral and sad faces relative to the HC group (*t’*s>3.26, *P*’s<0.005). The R-MD group had lower amplitudes for happy and neutral, but higher amplitudes for sad faces relative to the HC group (*t’*s>3.08, *P*’s<0.005). Also, the R-MD group had higher amplitudes for sad faces compared to the F-MD group (*t*(168) = 3.88, *P* = 0.004). In the HC subjects, amplitudes for happy faces were significantly higher than those of neutral and sad faces (*t’*s>2.73, *P*’s<0.008), and amplitudes for neutral faces were significantly higher than those for sad faces (*t*(91) = 2.89, *P* = 0.007). In both F-MD and R-MD groups, amplitudes for sad faces were significantly higher than those of happy and neutral faces (*t’*s>3.65, *P*’s<0.007), but no significant differences in amplitudes were found between happy and neutral faces (*P*’s>0.05) (Figure 2).

#### N170 latency

There was a significant main effect of group on longer latencies in the R-MD patients (mean = 201.62±28.16 ms). Latencies were longer when compared with those of the F-MD (mean = 192.07±31.42 ms) and the HC groups (mean = 173.85±21.09 ms) (*F* (2, 124) = 6.92, *P* = 0.025). A significant main effect of target expression was observed (*F* (2, 124) = 5.38, *p* = 0.031). No significant main effect of electrode location was found (*F* (1, 124) = 0.78, *P* = 0.126). The interaction of group×target expression was significant (*F* (2, 124) = 8.85, *P*<0.001).

Post hoc comparisons showed that F-MD and R-MD groups had longer latencies for happy, neutral and sad faces relative to the HC group (*t’*s>3.23, *P*’s<0.008), and the R-MD group had longer latencies for happy and neutral faces but shorter for sad faces compared with the F-MD group (*t’*s>3.60, *P*’s<0.006). In HC subjects, latencies for happy faces were significantly shorter than those of neutral and sad faces (*t’*s>3.02, *P*’s<0.010). In F-MD patients, none of the within-group comparisons showed significant differences (*P*’s>0.05). In R-MD patients, latencies for sad faces were significantly shorter than those of happy and neutral faces (*t’*s>4.23, *P*’s<0.004), but no significant differences in latencies were found between happy and neutral faces (*P*’s>0.05) (Figure 2).

### The relationship between HDRS_17_, behavioral performance, number of episodes and ERP data

**Figure 3:**
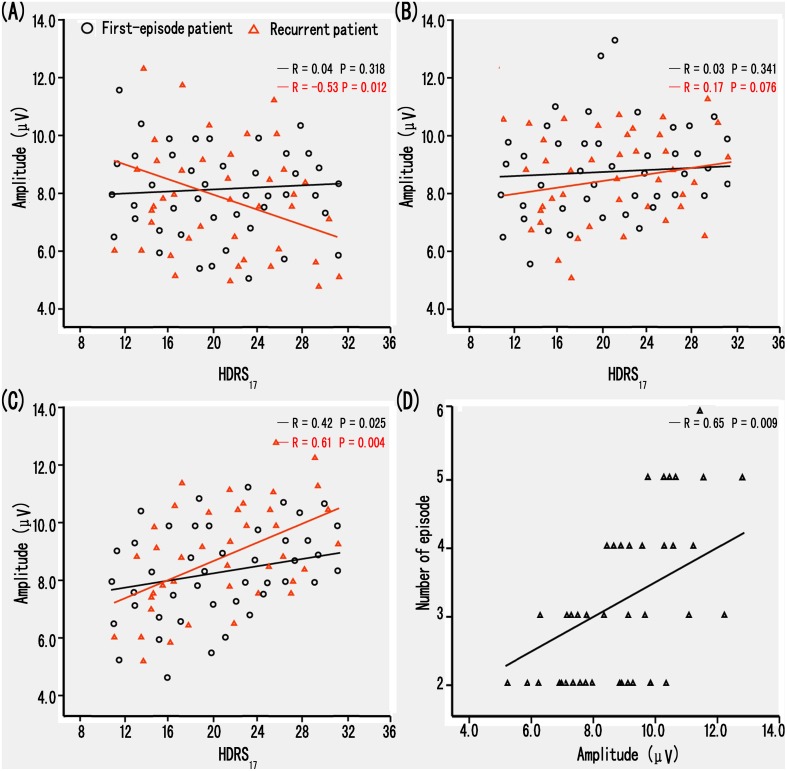
Correlation of clinical variables and the amplitude of event-related N170 potential in F-MD and R-MD patients. (A, B, C) Scattergrams representing the correlations between the groups and the N170 amplitude elicited by happy (A), neutral (B) and sad (C) facial pictures in F-MD (black line) and R-MD (red line) patients. (D) Scattergrams representing the correlations between the number of patient episodes and the N170 amplitude elicited by sad facial pictures in R-MD patients. Amplitude data are merged from P7 and P8 electrodes.

The multivariate regression analysis demonstrated that the deficits of N170 amplitude for happy and sad faces closely correlated with HDRS_17_ scores in R-MD patients (*F* (1, 39) = 12.02, *P*<0.001, negative relationship for happy faces; *F* (1, 39) = 16.92, *P*<0.001, positive relationship for sad faces) (Figure 3). The correlation between N170 amplitude for sad faces and HDRS_17_ score confirmed a markedly negative relationship in F-MD patients (*F* (1, 44) = 7.68, *P*<0.001) (Figure 3). However, no correlations were evident with respect to behavioral performance in the F-MD and R-MD groups (*P*’s>0.05). Interestingly, the multivariate regression analysis demonstrated that the deficits of N170 amplitude for only sad faces positively correlated with the number of episodes in R-MD patients (*F* (1, 39) = 14.36, *P*<0.001) (Figure 3). Additionally, no correlations were found between HDRS_17_ scores, behavioral performance, number of episodes or the deficits of N170 latency in the F-MD and R-MD groups (*P*’s>0.05). Furthermore, control subjects had no correlations between N170 index, behavioral performance or cognitive scores (all *P’*s>0.05).

## Discussion

To our knowledge, our study is the first to investigate the relationship between the abnormal neural processing of emotional facial expressions and the number of depressive episodes in patients. Our study further confirms that the electrophysiological processing of emotional facial expressions is altered in patients with depressive disorders. Our new data also provides new insights into understanding the unconscious negative cognitive bias, which may be an important biomarker for depression recurrence.

Our study reports that F-MD and R-MD patients had slower RTs and lower accuracies for identifying happy and neutral faces relative to HC subjects. This data suggests that their processing for happy and neutral faces is impaired. These results corroborated and expanded the previously described emotional processing deficits in clinical depression [41]–[43]. A noticeable exception was that R-MD patients had shorter RTs and higher accuracies for sad faces compared with the F-MD and HC groups. These results suggest that R-MD patients employ specific processing schemata for negative faces and rely on different processing mechanisms for the happy and neutral faces. In fact, R-MD patients are more excited for sad faces and may exaggerate the negative emotion [9], [44]. Taken together, the specific cognitive bias for sad faces by R-MD patients may be a consequence of focus on their inner world (negative emotion) [33], which may be strongly associated with interpersonal dysfunction in their clinical manifestation.

Our study also showed that F-MD and R-MD patients had reduced N170 amplitudes and longer N170 latencies for identification of happy and neutral faces. These data suggest dysfunctional emotional processing that includes the speed and strength of processing. These findings were also consistent with previous depression studies that showed impaired facial processing [4], [7], [45], [46]. Depressed patients have impaired performance with only positive stimuli [18]. Furthermore, patients with depressive disorders show reduced intensity and frequency of facial expressions with positive hedonic stimuli or specific physiological reactivity [47]. Several neuroimaging studies have also indicated that reduced amygdala activation in response to positive stimuli in depression may be linked to anhedonic symptoms caused by inappropriate salience attribution to positive information [48], [49]. Taken together, the evidence suggests that N170 impairment during facial processing may underlie one of the hallmark features of depression - anhedonic symptoms. It may also lead to a better understanding of interpersonal difficulties that are related to depression [42], [50], since patients utilize facial expressions as important indicators to manage their own behavior and to evaluate the attitudes of others.

Interestingly, our study indicated that R-MD patients had higher N170 amplitudes for sad faces compared to the F-MD and HC groups and shorter N170 latencies for sad faces compared with the F-MD group. This positive ERP in the R-MD group seems directly related to negative items and can be correlated with the bias for negative information [51], [52]. These findings were similar to previous depression reports that suggest faces expressing more sadness tend to be chosen as displaying greater intensity than happy and neutral faces. This may be due to the fact that sad faces are more arousing to R-MD patients than faces expressing other emotions when compared with the two other groups [52]. However, the negative cognitive bias was not found in F-MD patients, opposite of the R-MD patients who processed different emotional faces with a remarkably unconscious cognitive bias. This result suggests that R-MD patients have a specific emotional processing mechanism for negative faces, distinguishing them from F-MD patients. It further suggests that the unconscious negative cognitive bias may predict a sequential progression in F-MD patients, which could be strongly associated with the neuropathologic spread of depression. However, additional longitudinal studies will be needed to determine whether the unconscious negative cognitive bias in F-MD patients is specifically associated with the disease’s progression.

Our current study also investigated the relationship between HDRS_17_ score, behavioral data and ERP indexes. The data revealed a negative relationship between N170 amplitude and severity of depression for happy face recognition, while N170 amplitude was positively correlated with severity of depression for sad face recognition in R-MD patients. Collectively, these findings suggest that propagation of depression may intensify reductions in happy emotions while reinforcing negative emotions in R-MD patients. It is possible that the heightened perception of negative events may lead depressed individuals to feel worse or worthless about themselves and the whole outer world. Simultaneously, their lower mood can hinder their perception of happy events. Thus, they focus more on their inner world, which may be strongly associated with interpersonal dysfunction in their clinical manifestation [53]. However, only a negative relationship for sad face recognition was found in F-MD patients, suggesting that repeated episodes of depression may aggravate the deficits in emotional processing for happy faces and eventually lead to a lower mood [43]. Impaired positive emotional processing as indexed by N170 amplitude may be a useful and important biomarker of potential progression of depression [54].

In this study, the relationship between the amplitude and latency of N170 and the number of depressive episodes was also examined to confirm whether the unconscious negative bias in depression is a marker for stable cognitive vulnerability and possibly related to the recurrence of depression. Our results indicated that the N170 amplitudes for sad face recognition were significantly positively correlated with the number of depressive episodes, indicating that the emotional processing of sad faces differs with episode occurrence [4], [12]. As a result, it should be suggested that the hypersensitive perception of negative social stimuli is a stable cognitive vulnerability predictor associated with depression recurrence [7]. With episode repetition, patients generate specifically related mechanisms that result in negative bias reinforcement. The mechanisms can propagate depression through decreases in social reinforcement and social support that include negative feedback seeking, excessive reassurance seeking and interpersonal avoidance [4], [50]. Taken together, the evidence suggests that the unconscious negative cognitive bias may predict the recurrence of depression, and that the impaired negative emotional processing indexed by N170 amplitude may be a useful and important biomarker of potential depression recurrence.

There are, of course, limitations to this study. First, the sample size is relatively small in this study, which could affect the explanation of our results. Second, some of the depressed patients had mild anxiety symptoms, which could have influenced the results. Additionally, all patients were receiving antidepressant medication during the task. This could have influenced the behavioral or ERP performances of the patient groups. Finally, there are limitations imposed by the cross-sectional design of this study. Prospective longitudinal studies that assess changes in these complex relationships over time are needed. These future studies will help determine whether or not the changes in these parameters can be used as potential biomarkers for identifying individuals at high risk of recurrence and may clarify the diagnosis, or predict outcome in future studies.

## Conclusion

The current study provides novel insight into the relationship between the abnormal neural processing of emotional facial expressions and the number of patients’ depressive episodes. Our findings have important clinical implications for a more principled understanding of repeated physiopathological mechanisms of depression. Moreover, they further suggest that impaired emotional processing as indexed by N170 amplitude of positive and negative faces may be a useful biomarker for predicting the propagation and recurrence of depression. It will be a more significant and interesting contribution if future studies examine the relationship between the number of previous depressive episodes and the impaired emotional processing from the abnormal brain structure by functional magnetic resonance imaging technique.
