# Correction: Distinct Facial Processing Related Negative Cognitive Bias in First-Episode and Recurrent Major Depression: Evidence from the N170 ERP Component

**DOI:** 10.1371/journal.pone.0119894

**Published:** 2015-03-20

**Authors:** The PLOS ONE Staff

The eighth and ninth authors are listed out of order. The correct order is: Jiu Chen^1^, Wentao Ma^2^, Yan Zhang^2^, Xingqu Wu^2^, Dunhong Wei^2^, Guangxiong Liu^2^, Zihe Deng^2^, Zhijun Zhang^1^*, Laiqi Yang^2^*

1 Neurologic Department of Affiliated ZhongDa Hospital, Neuropsychiatric Institute and Medical School of Southeast University, Nanjing, Jiangsu Province, China,

2 Center for Mental Disease Control and Prevention, Third Hospital of the People’s Liberation Army, Baoji, Shaanxi Province, China

* E-mail: janemengzhang@vip.163.com (ZZ); yanglaiqi6666@163.com (LY)
